# Effects of *Bacillus subtilis* on in vitro ruminal fermentation and methane production

**DOI:** 10.1093/tas/txae054

**Published:** 2024-04-08

**Authors:** Efstathios Sarmikasoglou, Phussorn Sumadong, Gamonmas Dagaew, Mikayla L Johnson, James R Vinyard, Gerald Salas-Solis, Martha Siregar, Antonio P Faciola

**Affiliations:** Department of Animal Sciences, University of Florida, Gainesville, FL, 32611, USA; Department of Animal Science, Michigan State University, East Lansing, MI 48824, USA; Department of Animal Sciences, University of Florida, Gainesville, FL, 32611, USA; Department of Animal Science, Khon Kaen University, Khon Kaen 40002, Thailand; Department of Animal Sciences, University of Florida, Gainesville, FL, 32611, USA; Department of Animal Science, Khon Kaen University, Khon Kaen 40002, Thailand; Department of Animal Sciences, University of Florida, Gainesville, FL, 32611, USA; Department of Animal Sciences, University of Florida, Gainesville, FL, 32611, USA; Department of Animal Sciences, University of Florida, Gainesville, FL, 32611, USA; Department of Animal Sciences, University of Florida, Gainesville, FL, 32611, USA; Department of Animal Sciences, University of Florida, Gainesville, FL, 32611, USA

**Keywords:** direct-fed microbials, in vitro, methane

## Abstract

The objective of this study was to evaluate the effect of a proprietary strain of a *Bacillus subtilis* on in vitro ruminal fermentation and methane production in batch culture serum bottles. One hundred forty-nine batch culture bottles were used in a complete randomized block design. The arrangement of treatments was a 3 × 3 × 4 factorial to evaluate the effects of inoculum, time, diet, and their respective interactions. There were three experimental runs total, where the run was used as block. Inoculum treatments were 1.85 mg/mL of microcrystalline cellulose (CON); 10 billion *B. subtilis* plus microcrystalline cellulose (A1); and 60 billion *B. subtilis* plus microcrystalline cellulose (A2). Diet treatments were 0.50 g of early lactation diet (E, 30% starch), mid-lactation diet (M, 25% starch), or dry cow diet (D, 18% starch). The combination resulted in total of nine treatments. Each treatment had five replicates, two of which were used to determine nutrient degradability at 24 and 48 h after inoculation, and three were used to determine pH, ammonia nitrogen (NH_3_-N), volatile fatty acids, lactate, total gas, and methane production at 3, 6, 24, and 48 h after inoculation. Fixed effects of inoculum, diet, and their interaction were tested using the GLIMMIX procedure of SAS. Significance was declared at *P* ≤ 0.05. We observed that, compared to control, the supplementation of *B. subtilis*, decreased the production of acetate and propionate, while increasing the production of butyrate, *iso*-butyrate, valerate, *iso*-valerate, and caproate within each respective diet. Additionally, the total methane production exhibited mixed responses depending on the diet type. Overall, the inclusion of *B. subtilis* under in vitro conditions shows the potential to reduce ruminal methane production when supplemented with a mid-lactation diet, constituting a possible methane mitigation additive for dairy cattle diets.

## Teaser

*Bacillus subtilis* supplementation may decrease ruminal methane production when supplemented in a mid-lactation diet.

## Introduction

Approximately 62% of the total greenhouse gas emissions from the agricultural sector are produced by cattle and account for 3.8 gigatons CO_2_ -eq per annum (FAO, 2023). Ruminal fermentation is an important contributor of greenhouse gasses, primarily methane, originating from enteric fermentation in cattle (Macleod et al., 2013), thus several strategies have been developed to reduce methane emissions by modulating ruminal fermentation. Strategies to reduce methane production involve genetic selection, as well as dietary interventions. Feed additives, such as direct-fed microbials (DFM), 3-nitrooxypropanol, red seaweed, enzymes, and ionophores, (Hook et al., 2010; Hristov et al., 2013; Jeyanathan et al., 2014; Roque et al., 2021) have shown promising results. Direct fed-microbials such as *Bacillus subtilis* (*B. subtilis*) have been tested in previous studies, with observed potential to promote health and productivity of cattle (Kowalski et al., 2009; Ushakova et al., 2013; Lucey et al., 2021).

*Bacillus subtilis* is a nonpathogenic, Gram-positive, spore-forming bacterium (Piggot, 2009) that produces enzymes such as proteases, amylases, and cellulases with an evident role in improving gut health in humans (Rhayat et al., 2019) and enhances nutrient digestibility in livestock (Lucey et al., 2021). Previous studies have shown that *B. subtilis* could improve digestion at weaning (Ushakova et al., 2013), as well as, decrease the severity of diarrhea in calves (Kowalski et al., 2009). In addition, supplementation of *B. subtilis*, or with mannan-oligosaccharides has been reported to enhance passive immunity by increasing the average daily gain, and serum IgG levels at weaning in Holstein's calves (Sun et al., 2010; Lucey et al., 2021). Previous studies of *B. subtilis* in cattle have reported improvements in digestibility, performance, milk production, reduction in somatic cell counts, reduction in methane emissions, and stimulation of proteolytic and amylolytic bacteria growth (Peng et al., 2012; Sun et al., 2013; Jia et al., 2022).

Delivering DFM to the gastrointestinal tract of ruminants is challenged by the ruminal degradation, especially in cases where the DFM is in liquid or powder forms (Shen et al., 2017; Koh et al., 2022; Wang et al., 2022). Spherization, preceded by extrusion, has been reported to be an advantageous method over encapsulation, due to the formation of small-sized particles with a round shape with more uniform coating applications, thus increasing the potential for intestinal targeting (Muley et al., 2016; Koh et al., 2022; Rajam and Subramanian, 2022). Microcrystalline cellulose (MCC) is a commonly used excipient due to its unique binding properties, such as relatively low bulk density, high surface area, and high hygroscopicity (Thoorens et al., 2014). The major source of pharmaceutical MCC is purified from wood, where partially depolymerized cellulose is prepared by treating alpha cellulose with mineral acids (Thoorens et al., 2014).

Overall, the inclusion of DFM in feed is a common and well-established practice in the livestock industry that has improved the ruminal fermentation and overall cattle productivity (Duan et al., 2020; Lucey et al., 2021); however, the potential of *B. subtilis* in combination with MCC as carrier, has not been assessed yet. Therefore, the hypothesis of the present study was that *B. subtilis* conjugated with MCC would modulate ruminal fermentation towards a reduction in methane production and improve ruminal fermentation relative to feed degradation. Our objective was to determine the effects of *B. subtilis* conjugated with MCC on ruminal fermentation and methane production.

## Materials and Methods

### Ethical Approval

The University of Florida Institutional Animal Use and Care Committee approved all the procedures for animal care and handling required for this experiment.

### Experimental Design

One hundred forty-nine batch culture bottles were used in a complete randomized block design. Of the 149 bottles, 111 were used to determine the pH, total lactate, volatile fatty acids (**VFAs**), ammonia nitrogen (NH_3_-N), and gas production at 3, 6, 24, and 48 h after inoculation. Each treatment level had three replicate bottles and three blank bottles (rumen fluid only; to correct for background gas production). There were three experimental runs total, in which run was used as block. Samples were collected from a different set of bottles for each timepoint. The other 38 bottles were used for degradability measurements of DM (IVDMD) and NDF (IVNDFD). Each treatment had two replicates per treatment. There were three experimental runs total, where run was used as block. Samples were collected from a different set of bottles for each timepoint. The arrangement of treatments was a 3 × 3 factorial to evaluate the effects of inoculum, diet, and their respective interactions.

### Treatments

Three inoculums were used in this study. The inoculums were 1.85 mg/ mL of microcrystalline cellulose (CON); 10 billion *B. subtilis* plus microcrystalline cellulose (A1); and 60 billion *B. subtilis* plus microcrystalline cellulose (A2). Inoculums were supplemented in three different diets (early lactation diet, mid-lactation diet, and dry cow diet), resulting in nine treatments as they appear in Table 1. Dietary ingredients and chemical composition are indicated in Table 2. The concentrations of the two doses of *B. subtilis* were selected according to manufacturer guidelines and are comparable to the ones used in previous studies (Marenchino et al., 2023).

**Table 1.** Experimental treatments resulting from the combination of inoculums and diets used in a batch culture experiment with three different inoculum levels and three different diets

Inoculum^1^	Diet		
	Early	Mid	Dry
CON	E/C	M/C	D/C
A1 (10 billion cells)	E/A1	M/A1	D/A1
A2 (60 billion cells)	E/A2	M/A2	D/A2

^1^CON = 1.85 mg/ mL of microcrystalline cellulose, A1 = 10 billion *B. subtilis* plus microcrystalline cellulose, A2 = 60 billion *B. subtilis* plus microcrystalline cellulose.

**Table 2.** Ingredient and chemical composition of experimental diets used in a batch culture experiment with three different inoculum levels and three different diets

Item (% DM)	Early	Mid	Dry
Ingredient			
Corn silage	40	50	35
Bermuda grass hay	17	34	61
Ground corn grain	19	4	2
Soybean meal	24	12	2
Chemical composition^1^			
CP	18.8	14.7	11.7
EE	3.42	3.18	2.78
NDF	30.9	43.3	54.8
Starch	31.3	25.2	18.6
NEl (Mcal/kg)	1.69	1.48	1.25

^1^Expressed as a percent of DM unless otherwise stated.

Samples from feed ingredients, were ground through a 1 mm screen, and nutrient composition was determined. Those values were used as a reference for formulation of the experimental diets. Feed ingredients were ground through a 2 mm screen in a Wiley mill (model N°2; Arthur H. Thomas Co., Philadelphia, PA). Before grinding, corn silage was dried for 72 h at 60 °C in a forced-air oven (Heratherm, Thermo Scientific, Waltham, MA), until %DM was approximately 90% allowing for proper grinding and storage.

### Feeding and Management

Two ruminally cannulated Holstein cows in mid-lactation (108 ± 9.00 DIM on average) were used as ruminal inoculum donors. Cows were fed twice a day a total mixed ration with 38% corn silage, 19% ground corn, 13% soybean meal, 11% cotton seed, 9% citrus pulp, 8.5% mineral premix, and 1.5% palmitic acid supplement (on a DM basis) from 3 wk before the start and until completion of the experiment.

### In Vitro *Fermentation*

Approximately 3 h after morning feeding, ruminal contents were manually collected in equal proportions from each cow and strained through two layers of cheesecloth, transferred into pre-warmed thermoses, transported to the lab, and immediately mixed. The ruminal fluid used for incubations was then added to a buffered pre-warmed (39 °C) media (Goering and Van Soest, 1970) in a 1:2 ratio (rumen fluid: artificial saliva). The media was continuously infused with CO_2_ to maintain the anaerobic environment for the rumen fluid inoculum.

The in vitro experiment was conducted by using serum vials. Serum vials of 160 mL volume were used, containing 0.50 g DM of feed substrate and the corresponding inclusion rate for each treatment. Buffered rumen fluid (52 mL) was added to the 160 mL serum vials containing ANKOM bags with substrate or only substrate, and a continuous stream of CO_2_ was flushed into the vials during the whole inoculation process. The serum vials contained substrate either loose within the bottle for bottles used for fermentation characteristics or within a sealed ANKOM bag (F57; ANKOM Technology, Macedon, NY) for bottles used for degradability. Serum vials were closed with rubber stoppers and crimped with aluminum seals and then placed at 39 °C for 3, 6, 24, and 48 h in a mild shaking air-forced incubator.

### Sampling

Headspace gas (10 mL) was collected from each serum bottle at the respective timepoint (3, 6, 24, and 48 h), using a sealed gas injection needle, for determination of methane concentrations. After total gas and methane collections, the serum vials were opened, and pH was immediately measured using a portable pH meter probe (Thermo Fisher Scientific Orion Star A121, Waltham, MA, USA). Ten mL were collected and stored at − 20 °C from vials with substrate for later determination of total lactate, VFAs, and NH_3_-N. The IVDMD and IVNDFD degradability were estimated from the vials with the substrate pre-weighed into the ANKOM bags.

### Lactate Concentration

The ruminal fluid used for lactate analysis was first boiled at 100 °C for 10 min and then centrifuged at 10,000 × *g* for 10 min at 4 °C. The supernatants were collected and used for lactate analysis. The UV method was used for the determination of d- and l-lactic acid concentrations by using the d-lactic acid/l-lactic acid kit from R-Biopharm (R-Biopharm AG, Darmstadt, Germany). The analysis procedure was adapted from the manufacturer’s protocol. Specifically, 20 µL of sample, 80 µL of deionized water, 100 µL mix of glycylglycine buffer and l-glutamic acid, 20 µL of NAD, and 2 µL of glutamate-pyruvate transaminase suspension were loaded in a 96-well UV microplate, incubated at room temperature (RT) for 5 min, and read at 340 nm on a microplate spectrophotometer (Spectra Max 340 PC, Molecular Devices Corporation, Sunnyvale, CA, USA). Then, 2 µL of d-lactate dehydrogenase solution was added to each well and incubated at RT for 180 min and absorbance was read to determine the concentration of d-lactic acid. Then 2 µL of l-lactate dehydrogenase solution was added to each well and incubated in RT for another 180 min, and absorbance was read to determine the concentration of l-lactic acid. A 99% recovery of spiked concentration for both d- and l-lactic acid was considered as non-inhibitory dilution.

### VFA Analysis

Sample processing was done according to the study by Ruiz-Moreno et al. (2015). Briefly, the ruminal fluid used for VFA analysis was thawed at RT and centrifuged at 10,000 × *g* for 15 min; then the supernatant was mixed with crotonic acid and metaphosphoric acid solution and frozen overnight. The sample was then thawed at RT, centrifuged at 10,000 × *g* for 15 min and the supernatant was collected. The final supernatant was mixed with ethyl acetate (2:1; ethyl acetate: supernatant), vortexed, and allowed to settle with the top layer being transferred to a chromatography injection vial for analysis. Concentrations of acetate, propionate, butyrate, valerate, *iso*-butyrate, and *iso*-valerate in samples were analyzed via gas chromatography (Agilent 7820A GC, Agilent Technologies, Palo Alto, CA, USA) with a flame ionization detector and a capillary column (CP-WAX 58 FFAP, 25 m, 0.53 mm, Varian CP7767, Varian Analytical Instruments, Walnut Creek, CA, USA) where the temperature was maintained at 110 °C, with injector temperature at 200 °C and detector at 220 °C.

### Ammonia Nitrogen

Concentration of NH_3_-N in samples was analyzed according to Broderick and Kang (1980). Samples were thawed at RT and centrifuged at 10,000 × *g* for 15 min, and the supernatant was analyzed using the phenol-hypochlorite method in a 96-well flat-bottom plate. Absorbance was measured with a spectrophotometer (SpectraMax Plus 384 Microplate Reader, Molecular Devices, San Jose, CA, USA) at 620 nm.

### Chemical Analyses

The IVDMD and IVNDFD were calculated after 24 and 48 h of incubation, respectively. Pre-weighed ANKOM bags were taken out of serum vials, washed with tap water until effluent was clear and dried in a forced-air oven set at 60 °C for 48 h. Dried residue samples were analyzed for NDF using an Ankom Fiber Analyzer (Ankom Technology, Macedon, NY, USA). Residue weights and their NDF concentrations were used to calculate IVDMD and IVNDFD.

### Gas Pressure and Methane Concentrations

Gas pressure was measured at each timepoint using a pressure transducer (JYB-KO-M Pressure Transducer, Kunlun Tech Co Ltd, Beijing, China), for the determination of total gas concentration. Based on the conditions in the laboratory, the following equation was used to convert pressure to volume:


$$\begin{array}{l} Gas\;\;\;vol.\;(mL)=[Gas\;\;\;pressure\;(\;psi)*4.8843]+3.1296 \end{array}$$

Methane concentration was analyzed by gas chromatography (Agilent 7820A GC; Agilent Technologies, Palo Alto, CA). A flame ionization detector was used with a capillary column (Plot Fused Silica 25 m by 0.32 mm, Coating Molsieve 5A, Varian CP7536; Varian Inc. Lake Forest, CA). Injector, column, and detector temperatures were 80, 160, and 200 °C, respectively. Injector pressure was 20 psi with a total flow of 191.58 mL/min and a split flow of 185.52 mL/min with a 100:1 split ratio. Column pressure was 20 psi with a flow of 1.8552 mL/min. Detector makeup flow was 21.10 mL/min. The carrier gas was N_2_, and the run time was 3 min.

### Statistical Analyses

All data were statistically analyzed using the GLIMMIX procedure of SAS (SAS Institute Inc., Cary, NC). The statistical model used was as follows:


$$\begin{array}{lll} Y_{ijkl}= & \;\mu +\;I_{i}+D_{j}+H_{k}+I_{i}\times D_{j}+\;I_{i}\times H_{k}\; & +D_{j}\times H_{k}\;+\;I_{i}\times D_{j}\times H_{k}+E_{l}+\varepsilon_{ijkl} \end{array}$$

where, $Y_{ijkl}$, dependent variable, µ, overall mean; *I*_*i*_, inoculum; *D*_*j*_, diet; *H*_*k*_, hour; *E*_*l*_, experimental run; and $\varepsilon_{ijkl}$, random error. Experimental run was considered a random effect. Significance was declared at *P* ≤ 0.05 and a tendency was declared at 0.05 < *P* ≤ 0.10.

## Results and Discussion

The effect of diet was different (data not shown) among the treatments for all the tested variables; thus, all data were presented as the inoculum × diet, time × diet, and inoculum × time × diet for each respective diet.

### Effects on Ruminal Fermentation Characteristics

**Figure 1.**
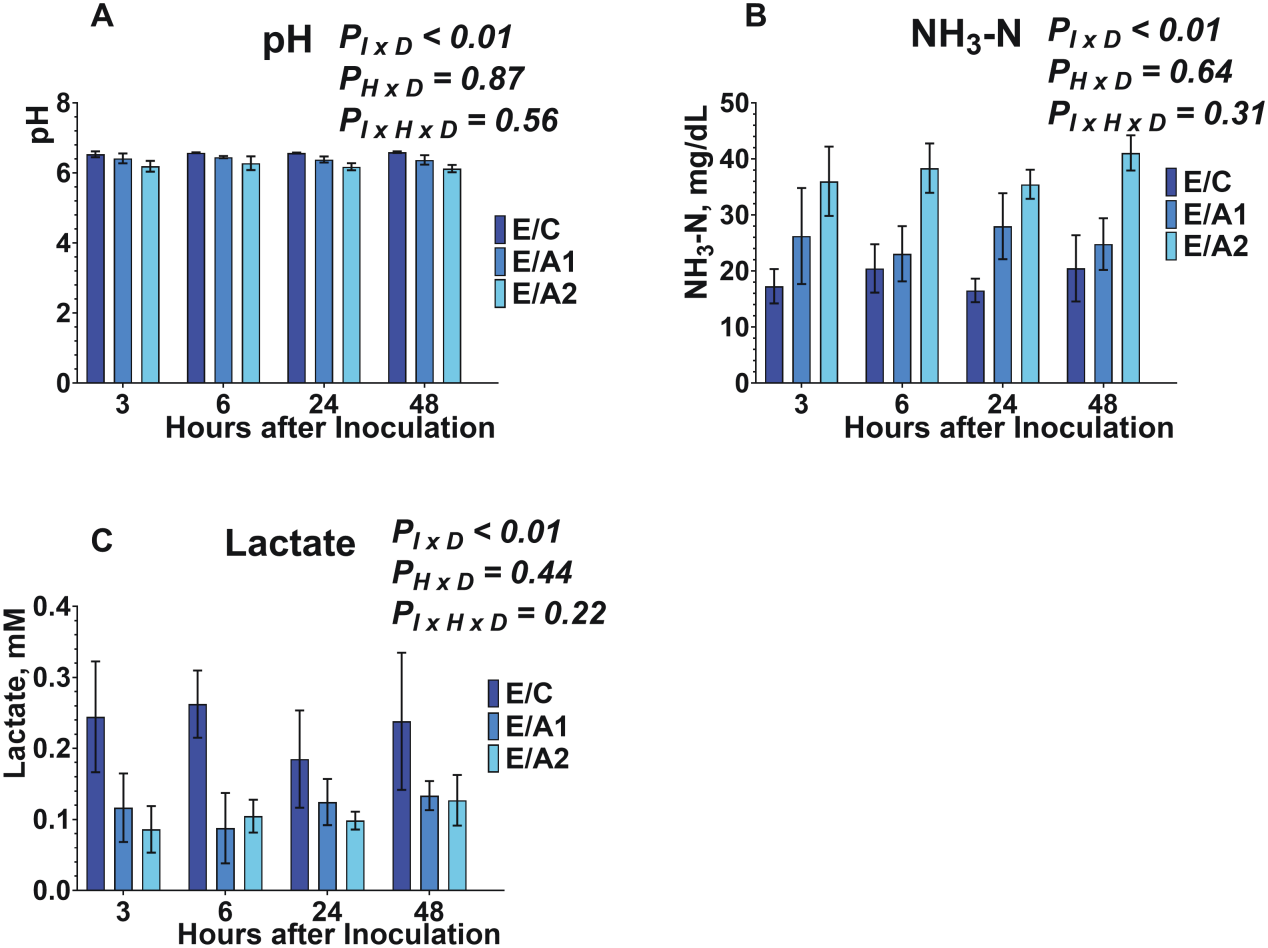
Effects of microcrystalline cellulose (CON), CON plus 10 billion *B. subtilis* (A1), and CON plus 60 billion *B. subtilis* (A2), supplemented with an early lactation diet (E) to buffered ruminal pH (1A), ammonia nitrogen (1B), and lactate production (1C), at 3, 6, 24, and 48 h after inoculation. Error bars refer to 95% CI.

**Figure 2.**
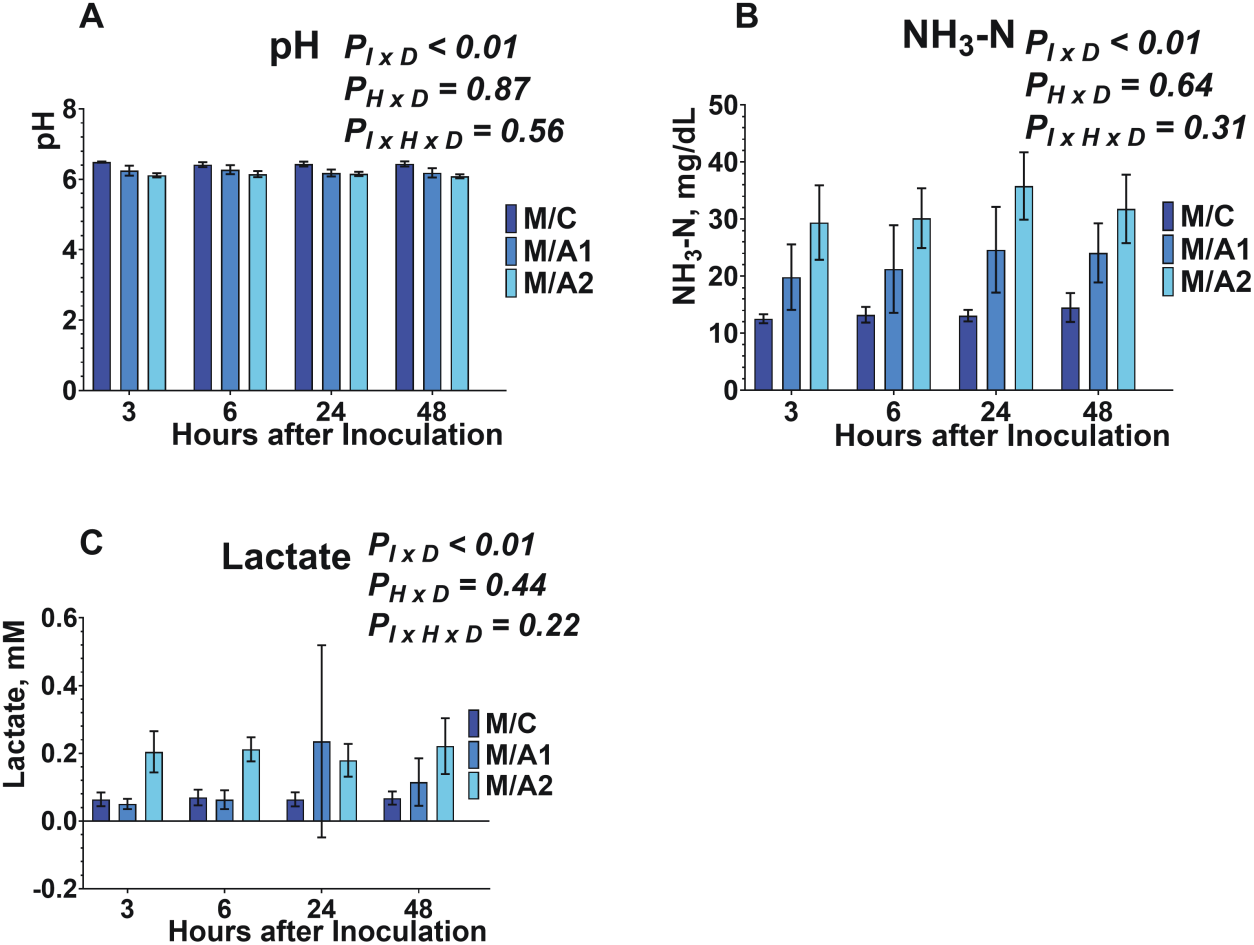
Effects of microcrystalline cellulose (CON), CON plus 10 billion *B. subtilis* (A1), and CON plus 60 billion *B. subtilis* (A2), supplemented with a mid-lactation diet (M) to buffered ruminal pH (2A), ammonia nitrogen (2B), and lactate production (2C), at 3, 6, 24, and 48 h after inoculation. Error bars refer to 95% CI.

Buffered ruminal fluid pH from serum vials was measured after 3, 6, 24, and 48 h of inoculation (Figure 1A, 2A, 3A). The inclusion of *B. subtilis* was different among the different diets tested (data not shown), and within each diet, there was an inoculum × diet (*P*_*I × D*_ < 0.01; data not shown) effect. No effect was exhibited on the time × diet (*P*_*H × D*_ = 0.87; data not shown), and the interaction of inoculum × diet × time (*P*_*I × H × D*_* *= 0.56). More specifically, ruminal fluid pH was greater (*P*_*I × D*_ < 0.01) in E/C, M/C, and D/C compared to either *B. subtilis* inclusion rates in each respective diet. Also, the E/A1, M/A1, and M/A1 exhibited greater (*P*_*I × D*_ < 0.01) ruminal fluid pH compared to E/A2, M/A2, and D/A2, respectively. Regarding the absence of an interaction between inoculum × time × diet, that indicates that the tested doses of *B. subtilis* affected ruminal fluid pH in a similar pattern to their carrier across time.

**Figure 3.**
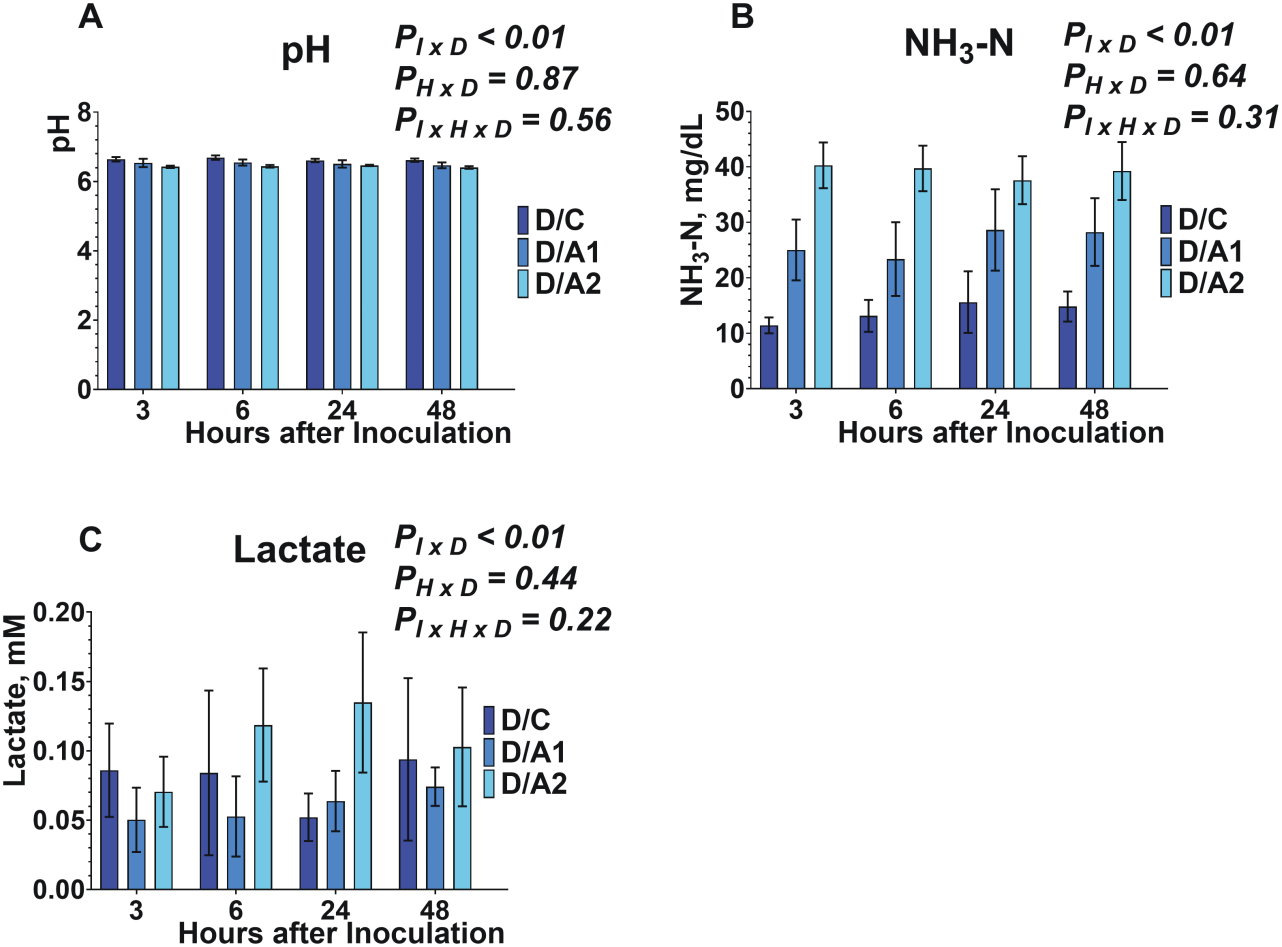
Effects of microcrystalline cellulose (CON), CON plus 10 billion *B. subtilis* (A1), and CON plus 60 billion *B. subtilis* (A2), supplemented with a dry cow diet (D) to buffered ruminal pH (3A), ammonia nitrogen (3B), and lactate production (3C), at 3, 6, 24, and 48 h after inoculation. Error bars refer to 95% CI.

Considering the MCC, previous studies have evaluated the inclusion of 10 and 100 g MCC/ kg DM to ruminal pH in multiparous Nordic Red dairy cows and found no effects (Savonen et al., 2020). In our study, the MCC treatment exhibited greater ruminal fluid pH, compared to the *B. subtilis* doses; however, at the different timepoints post-inoculation, there was no effect. The lack of effects from previous and our studies further indicates that the MCC would be an adequate carrier compound to supplement DFM in lactating dairy cow diets.

The buffering potential of DFM supplementation on ruminal pH has been previously highlighted (Martin and Nisbet, 1992; Ghorbani et al., 2002). Our data showed that in a 48 h fermentation, there was greater ruminal fluid pH in response to A2 inclusion, compared to A1 in each respective diet. However, the kinetics data suggest that there were no differences within each timepoint. In accordance with our study, previous studies reported no effects on ruminal pH from the inclusion of live *B. subtilis* or its fermentation products (proteases, and cellulases) in vitro (Chang et al., 2021) or in vivo (Peng et al., 2012; Sun et al., 2013; Lamontagne et al., 2023). Overall, our results suggest that *B. subtilis* and/or its metabolites are able to maintain the ruminal pH after 3, 6, 24, and 48 h, thus indicating a potential buffering activity. More specifically, the buffering potential of *B. subtilis* seems to protect from rapid ruminal pH drops due to the accumulation of organic acids in the rumen, incident that usually occurs under ruminal acidosis. Future studies should focus on investigating the sustained buffering potential of *B. subtilis* in the rumen.

The concentration of NH_3_-N was determined from serum vials after 3, 6, 24, and 48 h of inoculation (Figures 1B, 2B, 3B). Inclusion of *B. subtilis* was different among the different diets tested (data not shown), and within each diet, there was an inoculum × diet (*P*_*I × D*_ < 0.01) effect. No effects were exhibited on the time × diet (*P*_*H × D*_ = 0.64), and the inoculum × time × diet (*P*_*I × H × D*_* *= 0.31). More specifically, the concentration of NH_3_-N was lower (*P*_*I × D*_ < 0.01) in E/C, M/C, and D/C compared to either *B. subtilis* inclusion rates in each respective diet. Also, the E/A1, M/A1, and M/A1 exhibited lower (*P*_*I × D*_ < 0.01) NH_3_-N concentration compared to E/A2, M/A2, and D/A2, respectively. Regarding the absence of an interaction between inoculum × time × diet, that indicates that the tested doses of *B. subtilis* affected NH_3_-N concentration in a similar pattern to their carrier across time.

We used MCC as a carrier and our lack of effects in kinetics on NH_3_-N concentration are in agreement with previous studies that supplemented live 3.2 × 10^9^ cfu of direct-fed *Bacillus*, by using whey powder as carrier (Lamontagne et al., 2023). Lastly, the supplementation of MCC from unbleached softwood kraft pulp has been previously investigated with no effects in NH_3_-N concentration in vivo (Savonen et al., 2020).

In line with our study, supplementing 3.2 × 10^9^ cfu of direct-fed *Bacillus* (Lamontagne et al., 2023) or 6 to 12 g/d of *B. subtilis natto* fermentation metabolites (Peng et al., 2012) to multiparous lactating dairy cows did not change NH_3_–N concentration. Other studies supplemented 10^9^ cfu live *B. subtilis natto* with lactating dairy cow diets (Chang et al., 2021), or 10 to 20 g/ d of its fermentation metabolites (Sun et al., 2013), have reported an increase in NH_3_-N concentration. Overall, *Bacillus*-based cultures yield varied responses relative to NH_3_-N production, pointing to further research on their effect towards ammonia-producing and -utilizing bacteria in the rumen.

Lactate concentration was evaluated from serum vials after 3, 6, 24, and 48 h of inoculation (Figures 1C, 2C, 3C). Inclusion of *B. subtilis* was different among the different diets tested (data not shown), and within each diet, there was an inoculum × diet effect (*P*_*I × D*_ < 0.01; data not shown). No effect was exhibited on the time × diet (*P*_*H × D*_ = 0.44; data not shown), and the interaction of inoculum × diet × time (*P*_*I × H × D*_* *= 0.22). More specifically, lactate concentration was greater (*P*_*I × D*_ < 0.01) in E/C, compared to either *B. subtilis* inclusion rates in each respective diet. Also, the M/C exhibited greater (*P*_*I × D*_ < 0.01) lactate concentration compared to M/A2. Regarding the absence of an interaction between inoculum × time × diet, that indicates that the tested doses of *B. subtilis* affected lactate concentration in a similar pattern to their carrier during ruminal fermentation. To the best of our knowledge, this is the first study that evaluated the lactate concentrations in response to live *B. subtilis* supplementation in vitro, and the results indicate that diets with greater starch content would stimulate its metabolic rate. More specifically, *B. subtilis* can utilize starch as a carbon source (Ishikura et al., 1977; Schönert et al., 2006), thus diets with greater starch content would stimulate its metabolizable activity. However, the potential stimulatory effect seems to not be evident at specific timepoints during the fermentation.

**Figure 4.**
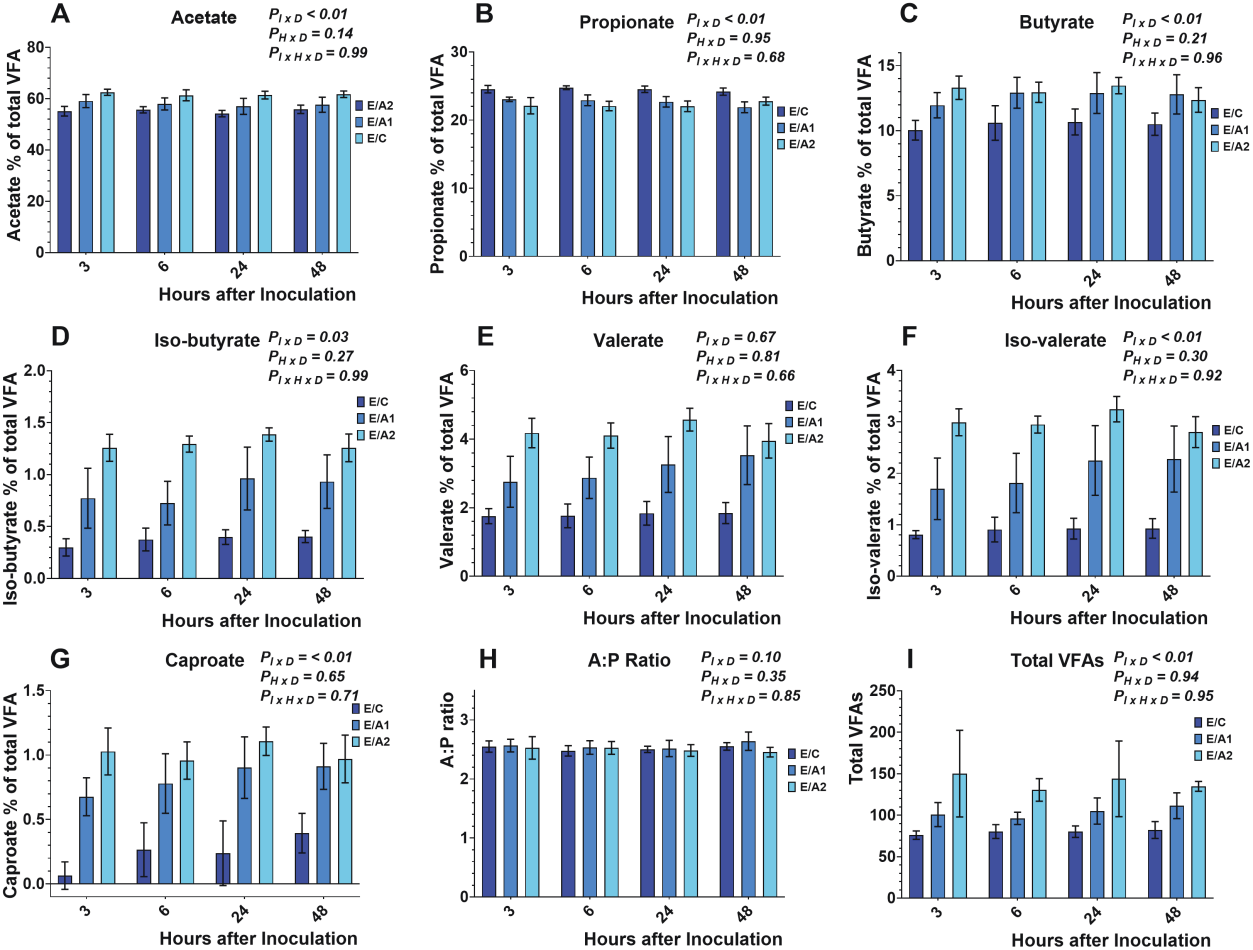
Effects of microcrystalline cellulose (CON), CON plus 10 billion *B. subtilis* (A1), and CON plus 60 billion *B. subtilis* (A2), supplemented with an early lactation diet (E) to acetate (4A), propionate (4B), butyrate (4C), *iso*-butyrate (4D), valerate (4E), *iso*-valerate (4F), caproate (4G), A:P; acetate to propionate ratio (4H), and total volatile fatty acids (4I) at 3, 6, 24, and 48 h after inoculation. Error bars refer to 95% CI.

**Figure 5.**
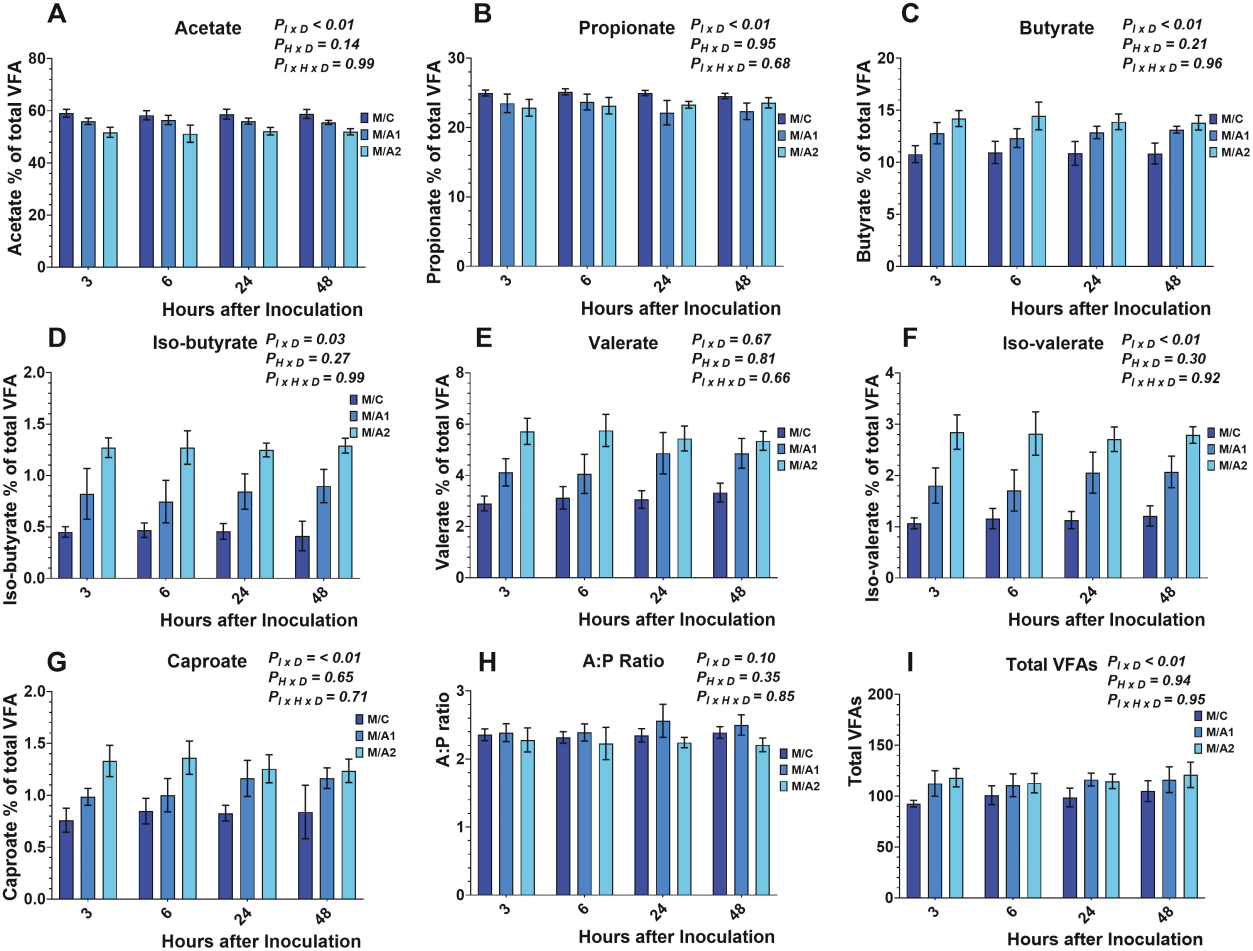
Effects of microcrystalline cellulose (CON), CON plus 10 billion *B. subtilis* (A1), and CON plus 60 billion *B. subtilis* (A2), supplemented with a mid-lactation diet (M) to acetate (5A), propionate (5B), butyrate (5C), *iso*-butyrate (5D), valerate (5E), *iso*-valerate (5F), caproate (5G), A:P; acetate to propionate ratio (5H), and total volatile fatty acids (5I) at 3, 6, 24, and 48 h after inoculation. Error bars refer to 95% CI.

**Figure 6.**
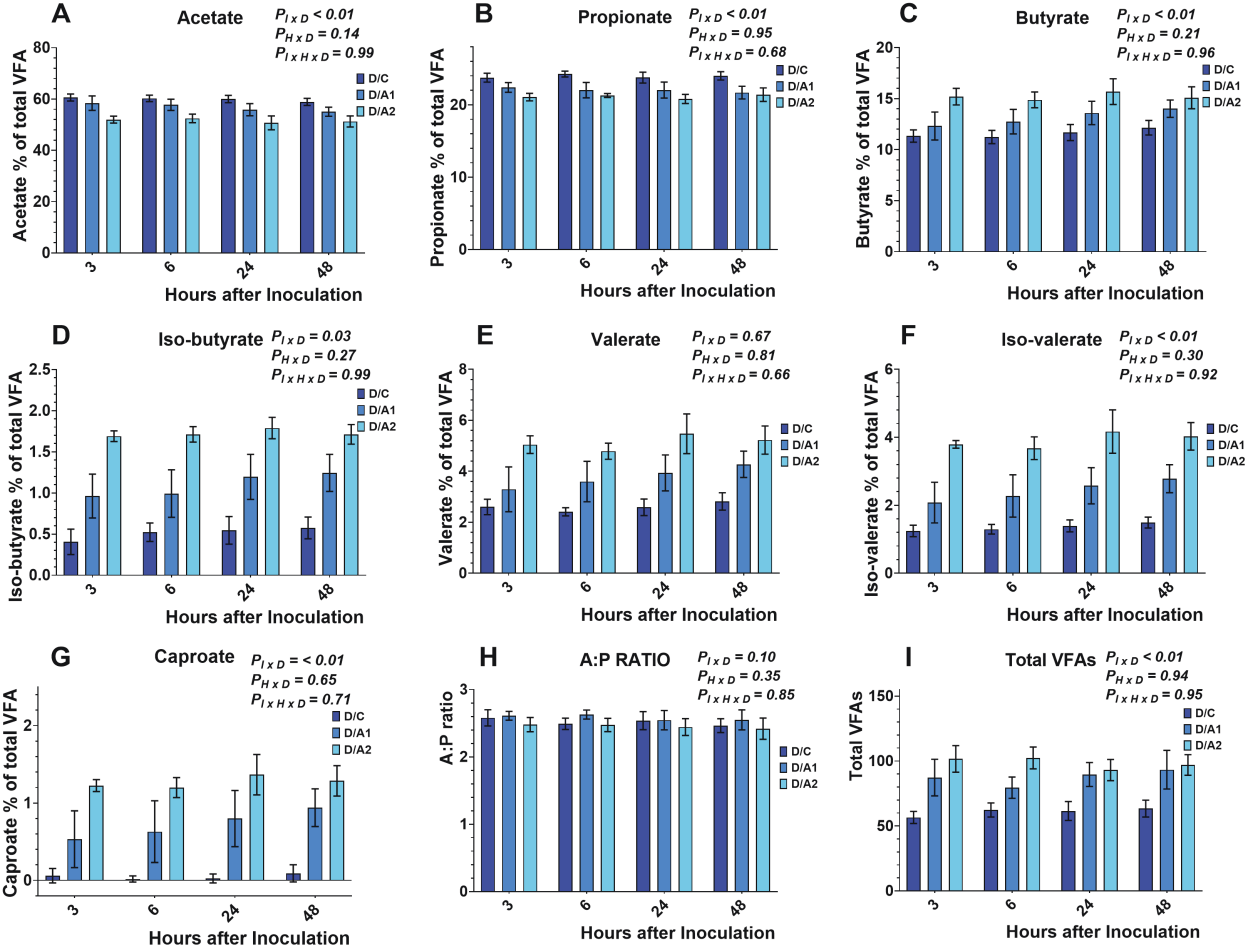
Effects of microcrystalline cellulose (CON), CON plus 10 billion *B. subtilis* (A1), and CON plus 60 billion *B. subtilis* (A2), supplemented with a dry cow diet (D) to acetate (6A), propionate (6B), butyrate (6C), *iso*-butyrate (6D), valerate (6E), *iso*-valerate (6F), caproate (6G), A:P; acetate to propionate ratio (6H), and total volatile fatty acids (6I) at 3, 6, 24, and 48 h after inoculation. Error bars refer to 95% CI.

Data on total VFA concentration and individual molar proportions are presented in Figures 4, 5, and 6. Total VFA concentration was different among the different diets tested (data not shown), and within each diet, there was an inoculum × diet (*P*_*I × D*_ < 0.01) effect. No effects were observed on the time × diet (*P*_*H × D*_ = 0.94), and the inoculum × time × diet (*P*_*I × H × D*_* *= 0.95). More specifically, total VFA concentration was lower (*P*_*I × D*_ < 0.01) in E/C, M/C, and D/C compared to either *B. subtilis* inclusion rates in each respective diet. Also, the E/A1 exhibited lower (*P*_*I × D*_ < 0.01) total VFA concentration compared to E/A2. Lower VFA production, in the absence of *B. subtilis*, can be explained by the greater ruminal fluid pH from the respective control treatments. However, the absence of inoculum × time × diet effect indicates that the tested doses of *B. subtilis* affected VFA production in a similar pattern to their carrier during the measured timespan.

Acetate molar proportion was different among the different diets tested (data not shown), and within each diet, there was an inoculum × diet (*P*_*I × D*_ < 0.01) effect. No effects were observed on the time × diet (*P*_*H × D*_ = 0.14), and the inoculum × time × diet (*P*_*I × H × D*_* *= 0.99). More specifically, acetate molar proportion was greater (*P*_*I × D*_ < 0.01) in E/C, M/C, and D/C compared to either *B. subtilis* inclusion rates in each respective diet. Also, the E/A1, M/A1, and D/A1 exhibited greater (*P*_*I × D*_ < 0.01) acetate molar proportion compared to E/A2, M/A2, and D/A2, respectively.

Propionate molar proportion was different among the different diets tested (data not shown), and within each diet, there was an inoculum × diet (*P*_*I × D*_ < 0.01) effect. No effects were exhibited on the time × diet (*P*_*H × D*_ = 0.95), and the inoculum × time × diet (*P*_*I × H × D*_* *= 0.68). More specifically, propionate molar proportion was greater (*P*_*I × D*_ < 0.01) in E/C, M/C, and D/C compared to either *B. subtilis* inclusion rates in each respective diet. Also, the D/A1 exhibited greater (*P*_*I × D*_ < 0.01) propionate molar proportion compared to D/A2.

Butyrate molar proportion was different among the different diets tested (data not shown), and within each diet, there was an inoculum × diet (*P*_*I × D*_ < 0.01) effect. No effects were exhibited on the time × diet (*P*_*H × D*_ = 0.21), and the inoculum × time × diet (*P*_*I × H × D*_* *= 0.96). More specifically, butyrate molar proportion was lower (*P*_*I × D*_ < 0.01) in E/C, M/C, and D/C compared to either *B. subtilis* inclusion rates in each respective diet. Also, the M/A1, and D/A1 exhibited greater (*P*_*I × D*_ < 0.01) butyrate molar proportion compared to M/A2, and D/A2, respectively.

*Iso*-butyrate molar proportion was different among the different diets tested (data not shown), and within each diet, there was an inoculum × diet (*P*_*I × D*_ = 0.03) effect. No effects were observed on the time × diet (*P*_*H × D*_ = 0.27), and the inoculum × time × diet (*P*_*I × H × D*_* *= 0.99). More specifically, the *iso*-butyrate molar proportion was lower (*P*_*I × D*_ < 0.01) in E/C, M/C, and D/C compared to either *B. subtilis* inclusion rates in each respective diet. Also, the E/A1, M/A1, and D/A1 exhibited greater (*P*_*I × D*_ < 0.01) *iso*-butyrate molar proportion compared to E/A2, M/A2, and D/A2, respectively.

Valerate molar proportion was different among the different diets tested (data not shown). No effects were exhibited on the inoculum × diet (*P*_*I × D*_ = 0.67), the time × diet (*P*_*H × D*_ = 0.81), and the inoculum × time × diet (*P*_*I × H × D*_* *= 0.66). More specifically, the valerate molar proportion was lower (*P*_*I × D*_ < 0.01) in E/C, M/C, and D/C compared to either *B. subtilis* inclusion rates in each respective diet. Also, the E/A1, M/A1, and D/A1 exhibited greater (*P*_*I × D*_ < 0.01) valerate molar proportion compared to E/A2, M/A2, and D/A2, respectively.

*Iso*-valerate molar proportion was different among the different diets tested (data not shown), and within each diet, there was an inoculum × diet (*P*_*I × D*_ < 0.01) effect. No effects were exhibited on the time × diet (*P*_*H × D*_ = 0.30), and the inoculum × time × diet (*P*_*I × H × D*_* *= 0.92). More specifically, the *iso*-valerate molar proportion was lower (*P*_*I × D*_ < 0.01) in E/C, M/C, and D/C compared to either *B. subtilis* inclusion rates in each respective diet. Also, the E/A1, M/A1, and D/A1 exhibited greater (*P*_*I × D*_ < 0.01) *iso*-valerate molar proportion compared to E/A2, M/A2, and D/A2, respectively.

Caproate molar proportion was different among the different diets tested (data not shown), and within each diet, there was an inoculum × diet (*P*_*I × D*_ < 0.01) effect. No effects were exhibited on the time × diet (*P*_*H × D*_ = 0.65), and the inoculum × time × diet (*P*_*I × H × D*_* *= 0.71). More specifically, caproate molar proportion was lower (*P*_*I × D*_ < 0.01) in E/C, M/C, and D/C compared to either *B. subtilis* inclusion rates in each respective diet. Also, the E/A1, M/A1, and D/A1 exhibited greater (*P*_*I × D*_ < 0.01) caproate molar proportion compared to E/A2, M/A2, and D/A2, respectively.

Previous studies that evaluated the effects of *Bacillus-*based DFMs on VFAs reported that 200 g/d/cow of *B. subtilis* or a mix of *B. subtilis* and *B. licheniformis* did not affect the molar proportion of acetate, propionate, and butyrate (Qiao et al., 2010), as well as, *iso*-butyrate, valerate, and caproate (Lamontagne et al., 2023). Moreover, other in vivo studies have found that 12 g/d/cow of *B. subtilis natto* fermentation product, increased propionate production (Peng et al., 2012; Sun et al., 2013), while others reported a decrease in acetate and an increase in valerate production (Sun et al., 2013). Lastly, in vitro, the inclusion of 10^9^ cfu live *B. subtilis natto* exhibited an increase in acetate, propionate, butyrate, valerate, and *iso*-valerate (Chang et al., 2021).

Overall, our study aligns with previous reports, showing a decrease in acetate (Sun et al., 2013), and an increase in butyrate, valerate, and *iso*-valerate (Chang et al., 2021) concentration by the inclusion of *Bacillus*-based DFMs. Interestingly, the reduction in propionate production with the inclusion of *B. subtilis* comes in contrast with previous studies; however, this study focused on the comparison of *B. subtilis* to its carrier and not a negative control, suggesting that the production pattern of propionate would be affected by the presence of MCC. Overall, *B*. *subtilis* cultures have exhibited conflicting responses on ruminal fermentation parameters indicating that further research should focus on elucidating their effect on specific groups of ruminal bacteria and consider the potential effect from their carriers.

**Figure 7.**
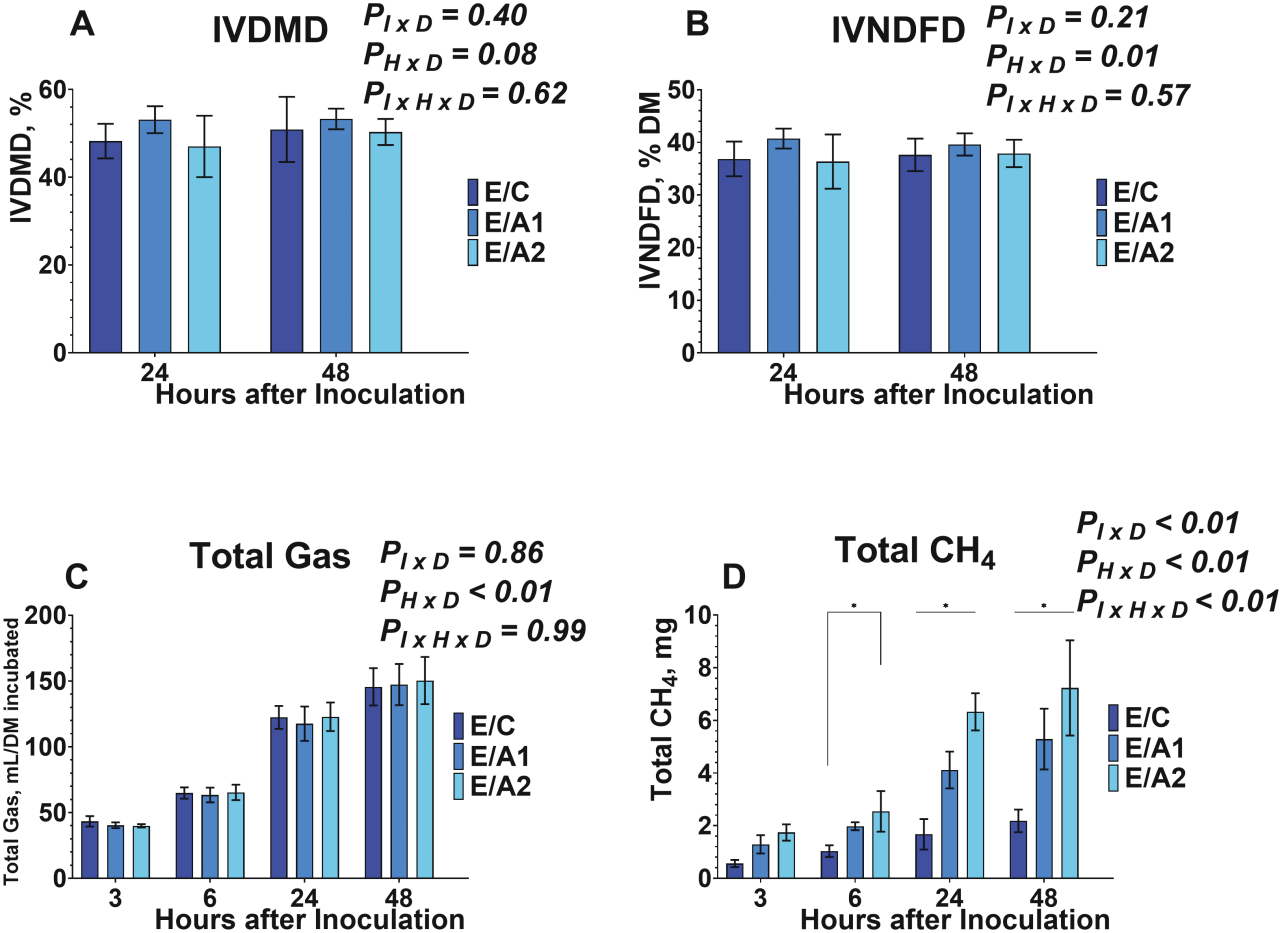
Effects of microcrystalline cellulose (CON), CON plus 10 billion *B. subtilis* (A1), and CON plus 60 billion *B. subtilis* (A2), supplemented with an early lactation diet (E) to in vitro dry matter degradability (7A), in vitro neutral detergent fiber degradability (7B), total gas (7C), and total methane (CH_4_, 7D) at 3, 6, 24, and 48 h after inoculation. Error bars refer to 95% CI.

**Figure 8.**
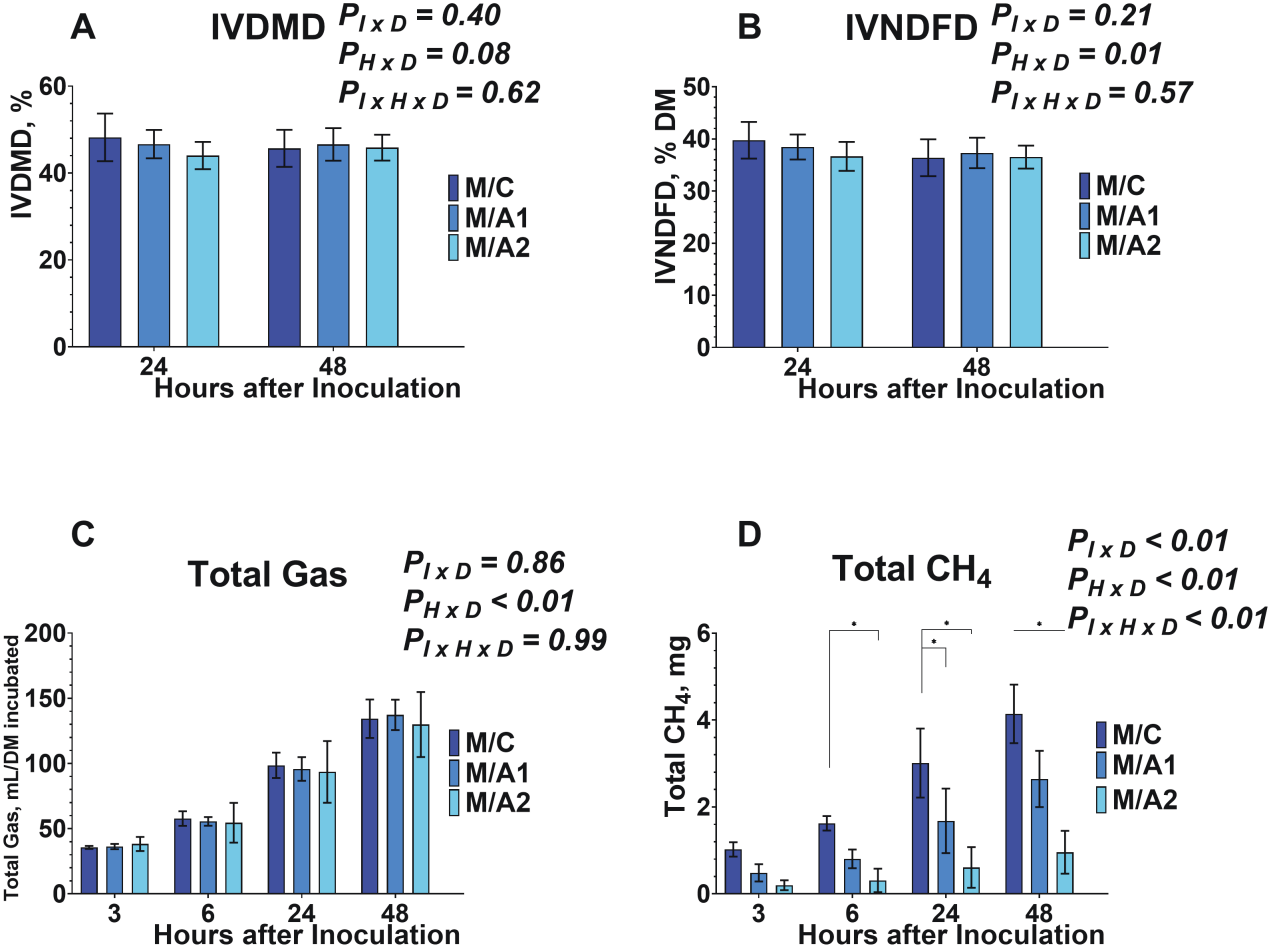
Effects of microcrystalline cellulose (CON), CON plus 10 billion *B. subtilis* (A1), and CON plus 60 billion *B. subtilis* (A2), supplemented with a mid-lactation diet (M) to in vitro dry matter degradability (8A), in vitro neutral detergent fiber degradability (8B), total gas (8C), and total methane (CH_4_, 8D) at 3, 6, 24, and 48 h after inoculation. Error bars refer to 95% CI.

**Figure 9.**
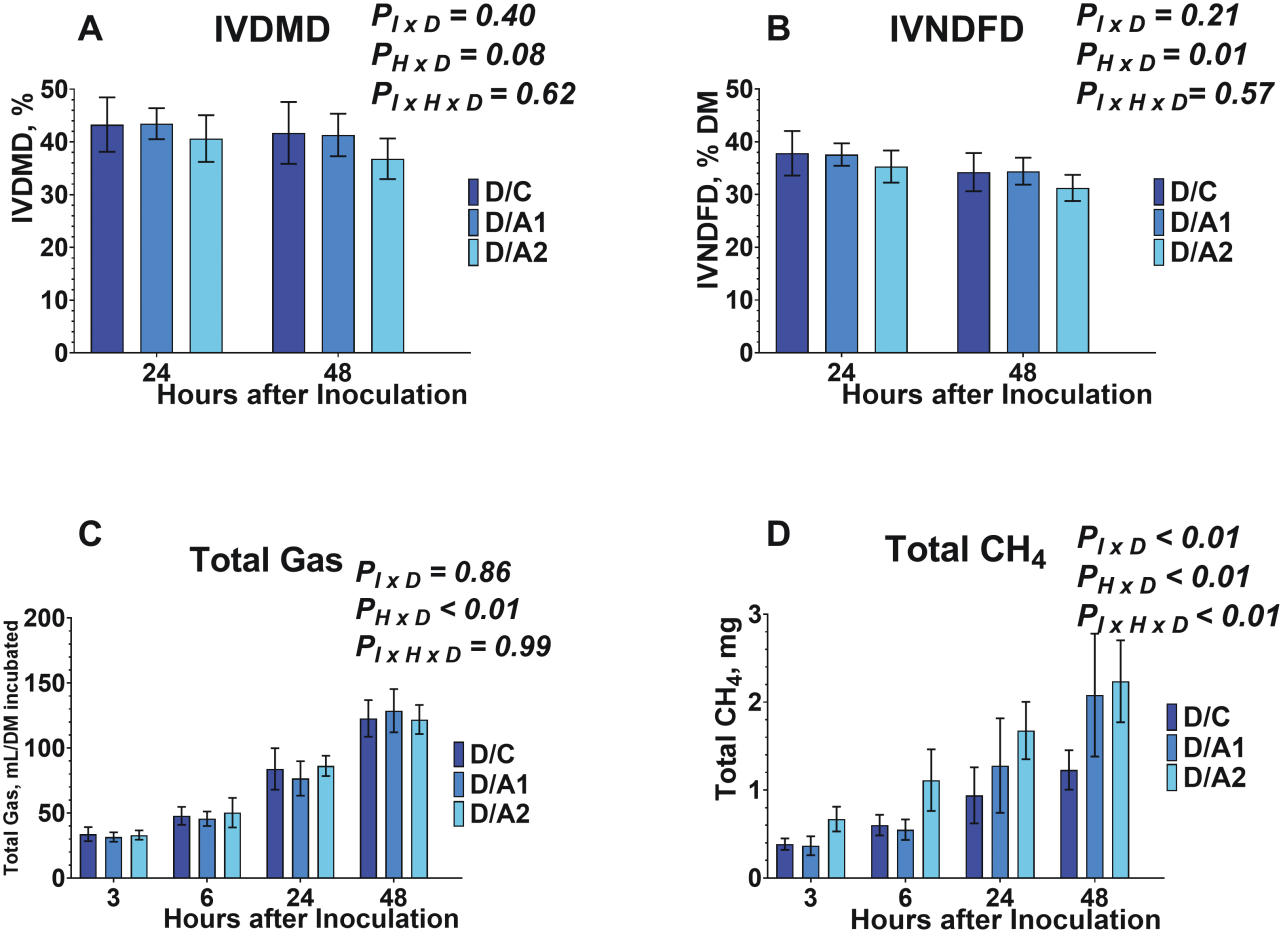
Effects of microcrystalline cellulose (CON), CON plus 10 billion *B. subtilis* (A1), and CON plus 60 billion *B. subtilis* (A2), supplemented with a dry lactation diet (D) to in vitro dry matter degradability (9A), in vitro neutral detergent fiber degradability (9B), total gas (9C), and total methane (CH_4_, 9D) at 3, 6, 24, and 48 h after inoculation. Error bars refer to 95% CI.

Regarding the IVDMD, and IVNDFD, both were evaluated from serum vials after 24 and 48 h of inoculation (Figures 7A and B, 8A and B, and 9A and B). The IVDMD was different among the different diets tested (data not shown). No effects were exhibited on the inoculum × diet (*P*_*I × D*_ = 0.40), on the time × diet (*P*_*H × D*_ = 0.08), and on the inoculum × time × diet (*P*_*I × H × D*_* *= 0.62). The IVNDFD was different among the different diets tested (data not shown), and on the time × diet (*P*_*H × D*_ = 0.01). No effects were exhibited on the inoculum × diet (*P*_*I × D*_ = 0.21), and the inoculum × time × diet (*P*_*I × H × D*_* *= 0.57). The lack of effects on inoculum × diet, and inoculum × time × diet for the degradability characteristics indicates that the presence and inclusion rate of *B. subtilis* are independent.

During ruminal fermentation, cellulose is hydrolyzed into cellobiose, which is later converted into pyruvate and finally into VFAs. Forage-to-concentrate ratios, as well as forage type in dairy cow diets, affect the rate of cellulose degradation and consequently the quantity of fermentation end products (Bampidis et al., 2020). Procedures such as the crystallinity of cellulose have resulted in decreased cellulose digestibility rate of up to 80% (Van Soest, 1994). In our study, the absence of effects aligns with previous reports that evaluated the effects of MCC as a dietary supplement in dairy cows (Savonen et al., 2020). These findings further support the fact that MCC would be utilized as a carrier substance to administer DFM. Regarding the effects of *Bacillus spp.* based on DFMs on digestibility, previous studies indicated a species-specific response. More specifically, most studies that supplemented *B. subtilis* (CJML_BIB_J_0011; Qiao et al., 2010; Sun et al., 2013; Souza et al., 2017; Jia et al., 2022) aligned with our data and found no effects, while others that supplemented *B. lincheniformis* exhibited a greater DMD (Qiao et al., 2010). Lastly, a previous study supplemented *B. subtilis natto* highlighted a lower NDFD despite the lack of effect on DMD, which would be potentially attributed to the activity of fibrolytic bacteria (Sun et al., 2013).

### Effects on Total Gas and Methane Production

Data on total gas are presented in Figures 7C, 8C, and 9C. Total gas was different among the different diets tested (data not shown), and on the time × diet (*P*_*H × D*_ < 0.01). No effects were exhibited on the inoculum × diet (*P*_*I × D*_ = 0.86), and the inoculum × time × diet (*P*_*I × H × D*_* *= 0.99). The absence of interaction between inoculum × diet, and inoculum × time × diet suggests that the tested doses of *B. subtilis* are affecting the total gas production in a similar pattern to their carrier.

Regarding the total methane production, data are presented in Figures 7D, 8D, and 9D. Total methane production was affected by the inclusion of *B. subtilis.* Differences were detected among the different diets tested (data not shown), different inoculums within each diet (*P*_*I × D*_ < 0.01). different timepoints within each diet (*P*_*H × D*_ < 0.01), and different timepoints and inoculums within each diet (*P*_*I × H × D*_ < 0.01).

More specifically, total methane production was lower (*P*_*I × D*_ < 0.01) in E/C compared to either *B. subtilis* inclusion rates in early lactation diet. Also, the E/A1, exhibited lower (*P*_*I × D*_ < 0.01) methane production compared to E/A2. In addition, the total methane production was greater (*P*_*I × D*_ < 0.01) in M/C compared to either *B. subtilis* inclusion rates in mid-lactation diet. Between M/A1 and M/A2, the higher dose of *B. subtilis* exhibited lower total methane production. Lastly, between the D/C, and D/A2 treatments the latter exhibited greater total methane production. Regarding the inoculum × time × diet, at 6 h post-inoculation, methane production increased by 84.36% with E/A2 compared to E/C (Figure 7D). In mid-lactation diet, the total methane decreased by 166.37% in response to M/A2 compared to M/C (Figure 8D). At 24 h post-inoculation production was greater by 212.09%, on average in response to E/A1 and E/A2 compared to E/C, and by 53.78% in response to E/A2 compared to E/A1 (Figure 7D). In mid-lactation diet, the total methane was lower by 61.99% on average in response to M/A1 and M/A2, compared to M/C (Figure 8D). At 48 h post-inoculation production was greater by 187.03%, on average in response to E/A1 and E/A2 compared to E/C, and by 36.73% in response to E/A2 compared to E/A1 (Figure 7D). In mid-lactation diet, the total methane was lower by 56.53% on average in response to M/A1 and M/A2, compared to M/C (Figure 8D).

Previous studies have evaluated the anti-methanogenic potential of *Bacillus-*based DFMs (Qiao et al., 2010; Wang et al., 2016; Jia et al., 2022). More specifically, their function is based on promoting propionate production, which subsequently decreases the H_2_ availability for methane production (Jeyanathan et al., 2014). Previous studies reported that supplementation with *B. subtilis* exhibited no effects (Qiao et al., 2010; Jia et al., 2022) or increase in total methane (Wang et al., 2016). In our study, the supplementation of *B. subtilis* with an early lactating dairy cow diet indicated an increase in total methane production similar to the findings of Wang et al. (2016), while no effects were observed when *B. subtilis* supplemented with a dry cow diet in agreement with the findings of the studies by Qiao et al. (2010), and Jia et al. (2022). On the other hand, when *B. subtilis* was supplemented a mid-lactation dairy cow diet, exhibited a decrease in total methane production, without an increase in propionate. The increase in propionate from the control groups in all diets leads us to believe that the *B. subtilis* would either directly inhibit rumen methanogens and/or inhibit specific rumen bacteria that produce methyl-containing compounds that are substrates for methanogenesis. Therefore, further research should focus on the effect of *B. subtilis* on inhibiting ruminal microbes associated with methanogenesis.

## Conclusions

In conclusion, compared to control, the supplementation of *B. subtilis*, decreased the production of acetate, and propionate, while increasing the production of butyrate, *iso*-butyrate, valerate, *iso*-valerate, and caproate within each respective diet. Additionally, the total methane production exhibited mixed responses depending on the diet type, which indicates that its function is diet specific and further research should focus on assessing its anti-methanogenic potential in cows in mid-lactation. Overall, future continuous culture, and in vivo studies are needed to further validate *B. subtilis* efficacy in modifying rumen fermentation.
